# Risks and Preventions for Pregnant Women and Their Preterm Infants in a World with COVID-19: A Narrative Review

**DOI:** 10.3390/vaccines11030640

**Published:** 2023-03-13

**Authors:** Abdulrahman K. Ahmed, Victor Coll Sijercic, Reem Sayad, Gregory R. Ruthig, Sayed F. Abdelwahab, Mohamed A. El-Mokhtar, Ibrahim M. Sayed

**Affiliations:** 1 Faculty of Medicine, Assiut University, Assiut 71515, Egypt; 2 North Central College, Naperville, IL 60540, USA; 3 Department of Biology, North Central College, Naperville, IL 60540, USA; 4 Department of Pharmaceutics and Industrial Pharmacy, College of Pharmacy, Taif University, P.O. Box 11099, Taif 21944, Saudi Arabia; 5 Department of Medical Microbiology and Immunology, Faculty of Medicine, Assiut University, Assiut 71515, Egypt; 6 Microbiology and Immunology Department, Faculty of Pharmacy, Sphinx University, Assiut 71515, Egypt; 7 Department of Biomedical and Nutritional Sciences, University of Massachusetts Lowell, Lowell, MA 01854, USA

**Keywords:** pregnancy, preterm birth, delivery, vertical transmission, COVID-19 vaccines

## Abstract

(1) Background and Aim: The severe acute respiratory syndrome coronavirus 2 (SARS-CoV-2) is linked to increasing cases of coronavirus disease 2019 (COVID-19) around the world. COVID-19 infections have an important impact on pregnancy, preterm birth (PTB) and delivery. Although several complications have been reported in infected pregnant women, the effect of infection on PTB is controversial. The purpose of this study was to summarize the existing literature on the effects and complications of COVID-19 on the health of pregnant women and preterm babies and its impact on the incidence of PTB. We also discuss the effect of current COVID-19 vaccines during pregnancy. (2) Methods: We carried out a systematic search of MEDLINE, Embase, and PubMed for studies on preterm births associated with COVID-19. (3) Results and Conclusions: We discovered contradictory results regarding the prevalence of PTB during the pandemic compared to earlier years. While most studies indicated an increase in PTBs with COVID-19, some indicated a decline in the preterm delivery rate during this time. During pregnancy, COVID-19 infection can increase the incidence of cesarean section, stillbirth, ICU admission, preeclampsia/eclampsia, and mortality rates. In the treatment of pregnant women with severe COVID-19, methylprednisolone was favored over prednisolone, and a brief course of dexamethasone is advised for pregnant women with anticipated PTB to accelerate the development of the fetal lung. Generally, vaccination for COVID-19 in pregnant and lactating women stimulates anti-SARS-CoV2 immune responses, and it does not result in any noteworthy negative reactions or outcomes for the mother or baby.

## 1. Introduction

Coronaviruses are positive-sense single-stranded RNA viruses circulating among humans and animals globally [1]. SARS-CoV-2 causes COVID-19, which has been associated with pandemics and fatalities since its initial characterization in Wuhan, China, in late 2019 [1,2,3]. A recent study reported that medical occupation does not impact the infection spread. Interestingly, females are slightly more likely to test positive than males [4]. Data on gender for the first 250 days of the COVID-19 pandemic in Bucharest, Romania showed that 53.2% of COVID-19 cases were females, and gender and age data should influence the prevention measures [5]. 

Positive cases have been on a continuous increase all over the world, among which pregnant women constitute an important group of cases [6]. During pregnancy, the chance of viral infection is considered to put both the pregnant mother and her fetus in danger. Some studies have reported increased incidence of preterm births (PTBs) with COVID-19 infection [6]. Pregnancy represents a special situation, since management decisions involve two patients: the mother and the fetus [7].

Pregnant women may experience significant COVID-19 expression due to the reduced lung volume associated with fetal growth and the suppression of the immune system during pregnancy [8,9]. Pregnant women who have COVID-19 have a higher risk of serious health problems such as ICU admission, mechanical ventilation, and PTB than non-infected pregnant women [9,10,11]. Premature membrane rupture, respiratory distress syndrome, preterm delivery, neonatal asphyxia, and neonatal mortality are among the more common obstetric problems that have been linked to known COVID-19 infection throughout pregnancy [12,13]. In this review, we focused on the impact of COVID-19 infection and the incidence of PTBs, the possibility of vertical transmission, and the types of COVID vaccines authorized for emergency use during pregnancy.

## 2. Preterm Delivery Rates: Did the COVID-19 Pandemic Cause an Increase or Decrease in Preterm Birth Rates?

Preterm delivery, also referred to as PTB or preterm labor, is parturition after 20 and before 37 weeks of gestation [14]. Recent studies on the incidence of PTB during the pandemic to earlier years revealed conflicting findings. Although most studies showed an increase in PTBs with COVID-19, several studies reported a decrease in the preterm delivery rate in the COVID-19 era (Table 1).

A report including 48 studies assessing the effect of COVID-19 infection on PTB <37 weeks that evaluated the effects of COVID-19 on pregnant women showed that 10.85% of infected mothers had a preterm birth, compared to only 6.0% in the control group (non-infected pregnant women). They acknowledged that it was challenging to determine whether preterm births were directly caused by COVID-19 or by a confluence of medical advice or responses to medications given to the woman to mitigate the disease’s effects, which might lead the data to lose rigor [9]. In another study, the proportion of preterm live births was higher among the infected women during pregnancy ratio (12.9%) compared to the general population of 2019 (10.2%), suggesting that the infection with SARS-CoV-2 may increase the risk of preterm birth, according to SET-NET, which included data from 16 jurisdictions [15]. Another study conducted in Romania reported an increase in 6.08% in PTB in women infected with COVID-19 (14.28%) compared with pre-pandemic levels (8.2%) and an increase in more than 13-fold in cesarean delivery from infected mothers compared to non-COVID-19 women [16].

Combining the results found in the Databases of Medline, Emblise, and Clinical Trials.gov, a 21.4% rate of PTB was observed in 2567 infected mothers with COVID-19 infection [17]. A report including 33 studies performed by Elshafeey et al. [18] found a 15.2% rate of PTB among infected women but did not compare their results to uninfected pregnant women. Smith et al. [19] surveyed the literature on PubMed, MEDLINE and EMBASE and documented a PTB rate of 63.8% among infected women and noted that the baseline rate of PTB in China was 6.7%. A study conducted on 132 women in a hospital in East India found a 15.4% increase in PTB in mothers infected with COVID-19 compared to the PTB rate in the preceding year [20]. A study focusing on COVID-19-infected pregnant patients hospitalized in 12 US institutions with critical outcomes found that 75% of women with critical disease suffered from PTB [21]. Patil et al. ran a cross-sectional study on 118 live births for SARS-CoV-2-infected women and showed that seven (16%) newborns were admitted to the NICUs after birth because of prematurity [22]. These findings were supported by many reports from the CDC showing increased PTB among women hospitalized during the SARS-CoV-2 pandemic [23,24].

However, other studies showed that PTB is decreased with COVID-19 infection [25,26,27,28,29]. In a study by Meyer et al., the maternal, obstetrical, and neonatal outcomes of singleton pregnancies at the Sheba Medical Center, Israel, were compared before and during the pandemic. They reported that the rate of PTB was significantly reduced by more than 50%, improving neonatal outcomes [26]. Another study conducted in the United States [25] reported a significant decrease in the PTB, especially in early and very early PTBs during the COVID-19 pandemic. In Denmark, a study of 31,180 live infants reported the probable effect of a countrywide lockdown on extreme PTB. Compared to the preceding five years, they discovered that the rate of extreme PTB dropped during the COVID-19 lockdown. They found that there were no significant differences in the rates of very preterm, moderate preterm birth, term, or post-term infants, which could indicate that such changes did not occur or that their incidence was too small to detect [27].

Another study in Ireland found that there was a 73% decrease in live births of very low birth weight (VLBW) infants and a 100% decrease in live births of extremely low birth weight (ELBW) infants compared to the previous 20 years [28]. Among the explanations proposed to explain the decrease in the PTB rate was a reduction in the level of stress, work, car driving, and air pollution as a result of the lockdown policy [25,26,27], avoidance of infections, reduced iatrogenic PTBs and the induction of HO-1, which may occur due to relative hypoxia caused by the mask mandates during the pandemic [26,27]. HO-1 increases hemoglobin synthesis and has been found to lower the rates of spontaneous PTB [26]. 

**Figure 1:**
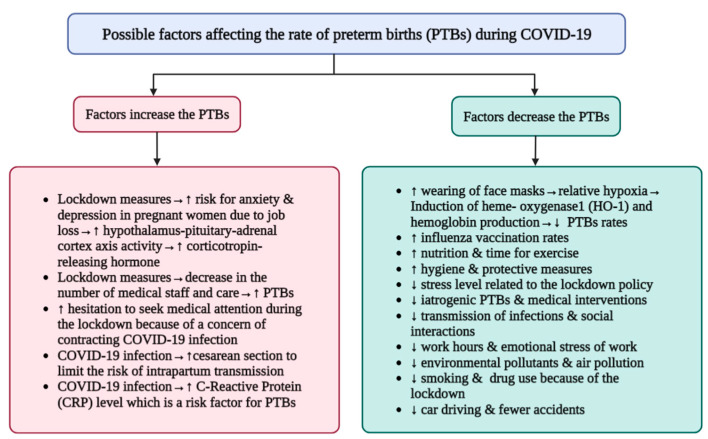
Various factors that could impact the frequency of preterm births (PTB) in the context of the COVID-19 pandemic. ↓ means decrease, reduction, ↑ means increase.

Most studies either reported a decrease in PTB rates or an increase in PTB rates, as indicated in Table 1, making our review article the first to explore the contradictory results of the influence of COVID-19 on PTB rates. In addition, we gathered most of the possible variables that could impact the PTB rate during COVID-19 (Figure 1) [9,15,16,17,20,21,22,23,24,25,26,27,28,29,30], providing information for further research. Further investigation is required to establish the precise causal relationship between COVID-19 and the rate of PTB, and the causative factors for this effect. This relationship can be translated into management that offers a successful strategy for the management of PTB associated with COVID-19-infected mothers and the associated morbidity and mortality.

**Table 1:** A summary of some studies reporting the outcomes and incidence of preterm deliveries in the era of COVID-19.

Author(s), Year	Study Type	Country	Study Subject	Outcomes	PTB Incidence	Ref
Allotey J et al., 2020	A systematic review and Meta-analysis	Global	Pregnant and recently pregnant women with COVID-19 (n = 293,152), and non-pregnant women with COVID-19 (n = 2,903,149)	Severe diseases are significantly increased in pregnant women with COVID-19 compared with healthy women, including PTB and maternal death.	Increased	[9]
Woodworth K et al., 2020	Cohort study	United States	Pregnant women with COVID-19 (n = 5252), Infants born to COVID-19-infected mothers (n = 3912)	Increase in 2.7% of PTB in pregnant women with COVID-19 compared to healthy individuals.	Increased	[15]
Bobei T et al., 2022	Prospective cohort observational study	Romania	Pregnant women infected with COVID-19 and having preterm birth (n = 34), healthy women having preterm birth (n = 48).	Strong positive correlation between PTB and cesarean section in mothers with COVID-19. Older COVID-19 pregnant women with PTB had more serious respiratory problems, lower oxygen saturation, higher inflammatory markers, and fewer lymphocytes.	Increased	[16]
Khalil, A et al., 2020	A systematic review and Meta-analysis	Global	Pregnant women with COVID-19 (n = 2567)	Increase iatrogenic PTB and cesarean delivery rates. Although rare, vertical virus transmission probably does happen in some instances.	Increased	[17]
Singh V et al., 2021	Retrospective Observational Study	East India	Pregnant women with COVID-19 (n = 132)	High rates of PTB and neonatal ICU hospitalizations. PTB occurred at a rate of 28.69%. The Caesarean section rate was 63.93%. Vertical transmission is feasible, although the probability is low. The rates of intrauterine and neonatal death remain low. Diabetes, hypertension, and anemia are comorbidity factors.	Increased	[20]
Pierce-Williams R et al., 2020	Cohort Study	United States	Hospitalized pregnant women with COVID-19 (n = 64)	PTB was found in 75% of women who had critical conditions of COVID-19. no evidence of vertical transmission, stillbirths, or neonatal deaths.	Increased	[21]
Patil, U.P et al., 2020	Retrospective cross-sectional study	United States	live births to mothers who had COVID-19 infection testing (n = 118)	16% of the infants were sent to NICU due to prematurity or possible sepsis. 11% of the infants were placed in an isolated room in early pandemic.	Increased	[22]
Villar, J et al., 2021	Paired Controlled Study	18 countries	Paired comparison between pregnant women with (n = 706) and without (n = 1425) COVID-19	Higher risk for preeclampsia/eclampsia, ICU admission, severe neonatal morbidity, and mortality index	Increased	[30]
Jafari M et al., 2021	A systemic review and Meta-analysis	Global	Meta Analysis of 228 studies of nonpregnant patients(n = 128,176) and 121 studies including pregnant patients (n = 10,000)	Increased caesarian delivery, low birth rate	Increased	[31]
Berghella V et al., 2020	Meta-Analysis	United States	Total births during COVID-19 (n = 1197), births before COVID-19 (n = 911)	25% reduction in PTB compared with pre-pandemic levels.	Decreased	[25]
Meyer R et al., 2021	Cohort Study	Israel	Group 1 (March 2020-June 2020): COVID-19 deliveries (n = 2594) Groups 2 (2019) and 3 (2011–2019), (n = 2742 and 28,686, respectively)	At < 34 0/7 weeks of gestation, the rate of PTB decreased by more than 50%.	Decreased	[26]
Hedermann G. et al., 2021	Nationwide prevalence proportion study (Observational Study)	Denmark	Live births born between March 12 and April 14, 2015-2020 (n = 31,180)	Significantly decreased rate of extreme PTB during the COVID-19 lockdown. The lockdown could reduce infant mortality and extreme PTB.	Decreased in extreme PTB	[27]
Philip R.K. et al., 2020	Retrospective descriptive study (Cohort Study)	Ireland	Live births of very low birth weight (VLBW) and extremely low birthweight (ELBW) infants from 1 January 2001 to 30 April 2020	A 100% decrease in ELBW infants and a 73% decrease in VLBW infants compared to the previous 20 years.	Decreased in VLBW and ELBW infants	[28]
Jasper B et al., 2022	Retrospective cohort study	Australia	Births born between April to July 2018–2020 (n = 64,989)	PTB dropped to 5.5% during the lockdown from 9.1% in previous years. Emergency Caesareans were reduced while instrumental vaginal deliveries increased during the lockdown.	Decreased	[29]

## 3. COVID-19-Infected Women Giving Birth during the Pandemic: Characteristics and Consequences

COVID-19 can increase the risk of serious maternal illness and prematurity. In addition, it can cause various serious symptoms and consequences, making it a very dangerous infectious disease, especially in pregnant women, who are a high-risk population (Table 2). The most frequent and serious complications are acute respiratory distress syndrome, septic shock and sepsis, acute heart and renal injury, sepsis, and other consequences [32]. Furthermore, severe pneumonia was also observed in a substantial percentage of pregnant patients, leading to the conclusion that pregnancy can increase the chances of a SARS-CoV-2 infection to progress into pneumonia [32]. Zambrano et al. ran a study on 409,462 symptomatic women who had COVID-19 with laboratory confirmation of whom 23,434 were pregnant women. Those symptoms ranged from mild fever, coughing, and chills, to an increase in ICU admissions [33]. COVID-19-positive pregnant women were substantially more likely to be admitted to an ICU in comparison to non-pregnant women. Although the actual risks of serious COVID-19-related complications in women were low, pregnant women had a markedly higher risk of serious complications than non-pregnant women [33].

A cohort study comparing the outcomes of women with COVID-19 who gave birth revealed a significant increase in the need for intubation, ventilation, ICU admissions, and mortality of infected mothers compared to healthy individuals. Chinn et al. studied a group of 869,079 adult women including 18,715 women with COVID-19. Infected women had a higher chance of mortality, PTB, and intubation in the hospital undergoing childbirth compared to non-infected women [34]. Similarly, a six-country retrospective study of women of reproductive age (n = 1315) who were admitted to hospitals during the COVID-19 pandemic showed that ICU admissions, oxygen therapy at admissions, and death were significantly higher in infected pregnant women compared to infected non-pregnant women and pregnant non-infected women [35]. Women with COVID-19 had a higher risk of stillbirth than women without COVID-19, particularly during the COVID-19 Delta variant predominance phase compared to the pre-Delta era [36]. DeSisto and colleagues conducted a study on 1,249,634 deliveries to assess the influence of COVID-19 on stillbirths. They observed a total of 8154 stillbirths throughout this period, impacting 0.64% of women without COVID-19 versus 1.26% of women with COVID-19 at delivery [36].

**Table 2:** Characteristics and outcomes of maternal COVID-19 infection.

Author(s), Year	Country	Study Subject	Outcomes	Ref.
Zambrano, L.D. et al., 2020	United States	Symptomatic COVID-19 women (n = 409,462), including pregnant women (n = 23,434)	↑ ICU admission and complications in pregnant women compared to non-pregnant women. Pregnant women had higher risk of acquiring COVID-19-related complications.	[33]
Chinn, J. et al., 2021	United States	Adult women (n = 869,079) including women with COVID-19 (n = 18,715)	↑ intubation, ventilation, ICU admissions, and mortality of infected COVID-19 mothers compared to non-infected ones.	[34]
Nachega, J.B. et al., 2022	Six countries in Sub-Saharan Africa	Women at reproductive age (n = 1315)	↑ ICU admissions, oxygen therapy at admissions, and death in infected pregnant women, compared to infected non-pregnant women and pregnant non-infected women.	[35]
DeSisto, C.L. et al., 2021	United States	Delivery hospitalizations (n = 1,249,634) including 21,653 deliveries to COVID-19 infected women.	↑ risk of stillbirth in COVID-19-infected, especially during the COVID-19 Delta variant predominance phase rather than the pre-Delta era than non-infected women.	[36]

↑ means increase.

## 4. Clinical Features and Short-Term Complications of Preterm Infants 

Pediatric SARS-CoV-2 infections could be asymptomatic or manifest with mild symptoms including headache, cough, nasal congestion, acute respiratory distress syndrome, anosmia, temperature instability, and runny nose [37,38]. This can be explained by the fact that the immune system of children is still developing, so they are unlikely to be able to initiate a cytokine storm like that of adults [39,40]. Dong et al. reported that confirmed pediatric patients with SARS-CoV-2 infection showed different degrees of manifestations, where 94.1% of cases were asymptomatic, mild, or moderate infections, and only 5.2% demonstrated severe manifestations [41]. Similarly, an outbreak that occurred in the United States in an overnight camp for children showed that 26% of COVID-19-infected children were asymptomatic [42].

Zhu et al. performed a study on ten neonates born to mothers who had tested positive for COVID-19. Six neonates developed shortness of breath, two neonates had fever, two neonates had thrombocytopenia and abnormal liver function, one neonate developed rapid heartbeat, one neonate had vomiting, and one neonate had pneumothorax [43]. In a cohort study conducted by Zeng et al. on 33 neonates born to COVID-19-positive women, three out of the 33 neonates tested positive for COVID-19. One of the three babies was reported as PTB. Due to fetal distress and maternal COVID-19 pneumonia, a second baby was delivered via cesarean section at 31 weeks and 2 days of gestation, and resuscitation was necessary. The infant required noninvasive ventilation, caffeine, and antibiotics for the treatment of pneumonia and neonatal respiratory distress syndrome. In addition, he exhibited leukocytosis, thrombocytopenia, and coagulopathy, as well as a blood culture that had come up positive for Enterobacter agglomerates [12].

**Figure 2:**
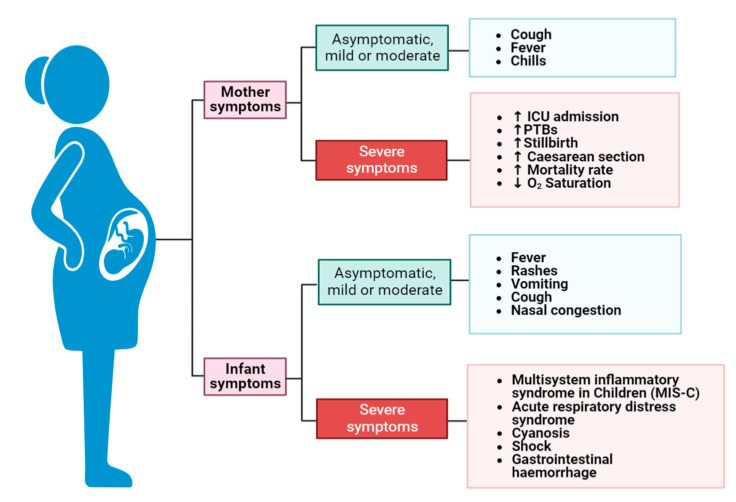
Complications and symptoms of COVID-19 during pregnancy. SARS-CoV-2 can affect both the pregnant woman and her infant. The symptoms might be unnoticed, mild, moderate, or severe, affecting both the mother and the infant’s lives. ↓ means decrease, reduction, ↑ means increase.

Recently, some reports have shown that COVID-19 can be associated with MIS-C in some infants. Some features of MIS-C in infants are similar to those of Kawasaki disease, while others are similar to those of toxic shock syndrome. Infants with MIS-C typically have a prolonged fever, gastrointestinal complications, and experience multisystem inflammation, with the cardiovascular system being the most affected [44]. These PTB correlated with a higher Body Mass Index (BMI) of the mother, cesarean delivery, and COVID-19 severity [45]. A study conducted on a cohort of 255 newborns of COVID-19-infected mothers revealed that COVID-19-exposed infants were at risk for both direct and indirect negative health consequences, while preterm delivery caused by a COVID-19 infection in the mother was linked to significant neonatal morbidity [46]. There was also a significant association between the rate of cesarean section and COVID-19 infection [45]. Various SARS-CoV-2 complications and symptoms that have been observed during pregnancy are shown in Figure 2. As shown, the symptoms might be absent, mild, moderate, or severe, affecting both the mother and the infant’s lives.

## 5. COVID-19 Vaccines: Safety and Efficacy during Pregnancy

One of the most important methods of protection during pregnancy is vaccination. Vaccines are used to prevent morbidity and mortality of the mother and to allow infants to acquire passive immunity [47]. Infants born to mothers vaccinated against smallpox during pregnancy are less liable to morbidity and mortality than infants of non-vaccinated mothers [47,48]. This is also true in the case of pertussis, tetanus, and influenza vaccination [47]. Because of the dangerous sequelae of COVID-19, especially in pregnant women, the need for vaccination is increased, but there have been worries about the side effects of vaccination [48]. Despite this, vaccination campaigns for pregnant women started while waiting for the results of clinical trials, and the campaign showed desirable results [49,50,51].

The first data about vaccination in pregnant women were related to Pfizer mRNA (BNT162b2) and Moderna (mRNA-1273) vaccines, which were used in the United States and Israel [50,51,52]. In one study, there were no observable safety concerns for mothers or infants from the vaccine-induced antibodies [52]. Among the reported effects recorded in the vaccinated subjects was injection-site pain; other symptoms such as fever, myalgia, chills, and headache were less frequent [51]. Of the vaccinated pregnant women who completed their pregnancy, 13.9% were pregnancy losses, and 86.1% were live births. According to neonatal outcomes, there was 9.4% PTB, and no neonatal death [51]. One of the most important results was that antenatal vaccination by BNT162b2 mRNA induces maternal hormonal responses that effectively transfer to the fetus, supporting the role of vaccination during pregnancy [53,54,55]. It was also reported that the vaccine is not associated with any pathological changes [54]. These data mean that COVID-19 vaccination is not associated with any developmental changes in the fetus, but immunological and inflammatory reactions occur at nearly the same rate as in the general population [52,56,57]. However, the probability that the placenta or fetus could be affected by an immunological reaction to the COVID-19 vaccination should be taken into consideration. Most safety data for these vaccines come from these results, which encouraged other countries to use them for pregnant women. Table 3 summarizes the types of COVID-19 vaccines authorized for emergency use during pregnancy and their side effects.

According to a recently published systematic review that summarizes the current data on COVID-19 vaccines including 23 studies and 117,552 COVID-19 vaccinated pregnant women [58], there was no difference in the obstetric outcomes between vaccinated and non-vaccinated pregnant women in the term of pre-eclampsia, placental abruption, pulmonary embolism, postpartum hemorrhage, ICU admission, or maternal death. Similarly, a systemic review by Fu et al. [59] concluded that vaccination for COVID-19 in pregnant and lactating women stimulated anti-SARS-CoV2 immune response, and did not result in any noteworthy negative reactions for the mother or the baby. Furthermore, it has been shown to be efficacious in preventing COVID-19 disease. Therefore, these results support the recommendations for the use of COVID-19 vaccine in pregnant mothers, because it does not increase the risk ratio in this category of the population.

Concerning vaccine efficacy, Piekos et al., in a retrospective, multicenter study, reported that 26,792 pregnant women who had received two doses of mRNA-1273 Moderna or BNT162b2 Pfizer/BioNTech vaccine and/or boosted (n = 7616) had a significantly lower risk of contracting the COVID-19 virus than matched pregnant women who had not received the vaccine. However, vaccination did not affect the duration of hospital stays for infected pregnant women, but it significantly reduced the percentage of patients receiving oxygen supply and vasopressor compared to those who did not receive the vaccination [60].

Studies revealed that the two groups (pregnant and nonpregnant women) had similar antibody and T-cell responses [56,61]. However, they also detected stronger virus-specific antibody titers linked to COVID-19 vaccination compared to SARS-CoV-2 infection, indicating that vaccines are still effective in people who have previously contracted the disease [56,61]. Another study reported that the overall antibody titers did not vary between pregnant women, lactating women, and non-pregnant groups, but that after only one dose of vaccine, Fc receptor binding and antibody effector functions were induced with delayed kinetics in the pregnant group in comparison to the non-pregnant group [62]. However, after the second dose, there was no significant difference between the groups [62].

In summary, COVID-19 vaccines decreased the rate of maternal COVID-19 infection among healthy pregnant women and reduced the need for supplemental oxygen supply and vasopressor among infected pregnant women, meaning that COVID-19 vaccines are effective even in those already infected with SARS-CoV-2.

**Table 3:** COVID-19 vaccines authorized for emergency use during pregnancy and the reported side effects.

Name	Pfizer/BioNTech	Moderna	SinoVac, Sinopharm	Bharat Biotech (Covaxin)	Serum Institute of India (Covishield)	Oxford/AstraZeneca	Janssen Biotech, Inc. (Johnson & Johnson)
Age	≥12	≥18					
Dosage	2 doses						Single dose
Dose interval	3 weeks	4 weeks	3–4 weeks	4–6 weeks	12–16 weeks	8–12 weeks	-
Type of vaccine	mRNA		Inactivated virus		Adenoviral vector		
Side effects	Local pain, redness, swelling, fatigue, headache, muscle pain, nausea and vomiting, and fever						
	Severe allergic reaction, myocarditis, and pericarditis		Diarrhea, cough, Joint pain, and allergies	Itching, rashes, and allergic reaction	Influenza-like illness, thrombocytopenia, and venous thrombotic events	Guillain-Barre syndrome, thrombosis with thrombocytopenia syndrome, and post-vaccination syndrome	
References	[2,10,58,63,64,65,66]						

## 6. Modes of COVID-19 Infection Transmission from the Mother to the Fetus

According to the most recent studies on the modes of SARS-CoV2 transmission, respiratory droplets, contact transmission, and aerosol transmission are the three main modes of transmission [67,68]. Additionally, SARS-CoV2 has been found in stool samples, so fecal–oral transmission could be another mode of transmission [67,68]. Although COVID-19 infections have been observed in pregnant women and newborns, vertical transmission remains a controversial issue [12,38,69,70,71,72]. Some cases have recently emerged as transplacental transmission due to placental involvement and neonatal infection was confirmed by RT-PCR in a nasopharyngeal (NP) swab at birth [73,74]. SARS-CoV-2 RNA was found in an NP swab sample obtained on the day of birth with no risk of contact with vaginal secretions or the skin of the mother, suggesting congenital transmission of the virus [75]. Similarly, high levels of IgM antibody in the fetus borne to the infected mother indicate intrauterine infection [76,77]. However, Kimberlin and Stagno pointed out that the presence of specific IgM in the newborn is inadequate to confirm the in-utero infection, and a false positive result is probable [78]. A recent systematic review included 936 newborns from COVID-19-infected mothers; 27 newborns had a positive RT-PCR using a NP swab, implying a pooled proportion of 3.2% for vertical transmission, suggesting that vertical transmission of COVID-19 is possible and appears to occur in a minority of cases of maternal COVID-19 infection in the third trimester [79].

SARS-CoV-2 was also detected in the breast milk of infected breastfeeding mothers [80,81]. Chambers et al. described the detection of viral RNA in the milk of one out of 18 infected mothers, but the culture was negative, indicating that the presence of viral RNA does not imply infectivity [82]. Still, the possibility of vertical transmission through breastfeeding needs further investigation. In a retrospective study by Chen et al. including pregnant women with COVID-19, breastmilk samples of all the tested samples were negative for SARS-CoV-2 [70]. Therefore, most pediatric guidelines do not warn against breastfeeding COVID-19 mothers [83]. Due to the increasing number of COVID-19 cases every day, a set of regulations has been established to limit the number of parents who reach the NICU to prevent the transmission of infection from parents to newborns, and this limitation influences the psychological and physiological health of both parents and newborns [84,85]. The WHO recommended not to separate mothers from newborns even if they are suspected, despite the transmission of SARS-CoV2 from mothers to fetuses through direct contact, breastfeeding, and respiratory droplets [86].

## 7. Prevention, Management, and NICU Admission of Preterm Infants of COVID-19-Infected Mothers

I Infection of pregnant women with COVID-19 had a minimal effect on preterm delivery and neonatal short-term outcomes when they were given an appropriate delivery procedure, good management, and efficient infection precautions [87]. If sufficient management is applied, the possibility of vertical transmission of SARS-CoV-2 in premature newborns to COVID-19-infected mothers is relatively low [87]. Infants born to mothers who have COVID-19 should be categorized as suspected patients who should be isolated and monitored [88]. A large number of guidelines advise that a newborn baby should be isolated in the specified NICU, but some permit rooming in the mother’s room, with suitable infection control measures [38,89]. The delivery room should be properly equipped and prepared when dealing with confirmed infected mothers, particularly with negative pressure, if available, and all the physicians should wear PPE [67].

COVID-19 treatments are mostly conservative for pregnant women who are expecting a premature birth. Antiviral medications and high doses of corticosteroids are rarely used, because of inconsistent efficiency and/or adverse effects in preterm infants [90]. However, the use of prednisolone or hydrocortisone is recommended for pregnant women with moderate to severe COVID-19 [91]. Vardhelli et al. recommended the use of antenatal steroids and magnesium sulfate for protection against perinatal COVID-19 [92]. A study conducted on 81,832 newborns who were born following 23 to 34 weeks’ gestation and exposed to prenatal corticosteroids showed that the therapy decreased mortality and morbidity compared to no exposure [93]. When treating pregnant women with severe COVID-19, methylprednisolone was preferred over prednisolone in treating pregnant women with severe COVID-19 [91]. Additionally, a brief course of dexamethasone is recommended for pregnant women with expected PTB to speed up the development of the fetal lung [91].

COVID-19-positive newborns should be nursed in an incubator and transferred to the NICU [92]. Negative-pressure isolation rooms or rooms with high-efficiency particulate air filters are suitable for the neonate. Staff can manage the baby with isolation precautions and PPE, and they should not be moved to other parts of the NICU [92]. NICU should only be used for neonates who need intensive care to decrease the number of required beds and avoids putting critical and emergency cases in a dangerous situation [92,94,95].

## 8. Conclusions

SARS-CoV2-infected pregnant women suffer higher rates of severe disease than non-pregnant infected women and higher rates of stillbirths than non-infected women. While children generally have mild COVID-19 symptoms, infants born to infected women were at risk for negative health consequences. Whether COVID-19 causes preterm births is unclear, with conflicting results from different studies around the world. Prednisolone or hydrocortisone were administered to decrease morbidity and mortality and antenatal steroids and magnesium sulfate were recommended to protect against perinatal COVID-19. It remains unclear if women can pass SARS-CoV2 to their offspring via the placenta or breast milk and infected women are recommended to remain with their newborns after birth. Preterm babies that do need to be cared for in the NICU should be housed in negative pressure rooms with minimal exposure to non-infected infants.

## Data Availability

Not applicable.

## Abbreviations

CDC: Centers for Disease Control and Prevention, ELBW: Extremely Low Birth Weight, HO-1: heme-oxygenase1, ICU: Intensive Care Unit, MIS-C: Multisystem Inflammatory Syndrome in Children, NICU: Neonatal Intensive Care Unit, NP: Nasopharyngeal, PPE: Personal Protective Equipment, PTB: Preterm Birth, RT-PCR: Reverse Transcriptase–Polymerase Chain Reaction., SET-NET: Surveillance for Emerging Threats to Mothers and Babies Network, VLBW: Very Low Birth Weight, WHO: World Health Organization.
